# Southern Medical Journals

**Published:** 1865-11

**Authors:** 


### Southern Medical Journals.

*Richmond Medical Journal.*—We are gratified to be able to announce the probable appearance of the *Richmond Medical Journal*, the first number of which will appear in December next. It will be esited by Dr. E. S. Gaillard, who is widely and favorably known as an accomplished scholar, skillful physician and a ready and forcible writer. This journal is to be a monthly octavo, of from 80 to 90 pages, and will be published at $5 00 a year, in advance. We bespeak for the journal a liberal support from the profession.

*The Medical and Surgical Monthly*, of Memphis, Tenn., is also proposed for the new year. It is to be edited by Frank A. Ramsey, A. M., M. D., a physician of high literary and professional attainment. It is to be published at the subscription price of $6 00 per year, and to contain 64 pages, each number. We hope that with restored peace, may again return in the South an active cultivation of Medical Science.

*Lindsay and Blakiston's Physicians' Visiting List, and Book of Engagements for* 1866.—The profession are now fully aware of the convenience of this Pocket Companion. The publishers deserve many thanks for furnishing so compact, convenient and valuable a book; one which answers so many purposes, and at so small an outlay. For sale in Buffalo by THEODORE BUTLER, 159 Main St.

